# Clinical validation of novel lightning dose optimizer for gamma knife radiosurgery of irregular‐shaped arteriovenous malformations and pituitary adenomas

**DOI:** 10.1002/acm2.13669

**Published:** 2022-06-24

**Authors:** Damodar Pokhrel, Mark E. Bernard, James Knight, William St. Clair, Justin F. Fraser

**Affiliations:** ^1^ Department of Radiation Medicine Gamma Knife Radiosurgery Center University of Kentucky Lexington Kentucky USA; ^2^ Departments of Neurological Surgery, Neurology, Radiology, and Neuroscience University of Kentucky Lexington Kentucky USA

**Keywords:** AVMs and pituitary adenomas, forward planning, gamma knife SRS, inverse planning, lightning dose optimizer

## Abstract

**Purpose:**

To demonstrate the clinical feasibility of a novel treatment planning algorithm via lightning dose optimizer (LDO) on Leksell Gamma Knife (LGK) GammaPlan with significantly faster planning times for stereotactic radiosurgery (SRS) of the complex and difficult arteriovenous malformations (AVMs) and pituitary adenomas.

**Methods and materials:**

After completing the in‐house end‐to‐end phantom testing and independent dose verification of the recently upgraded LDO algorithm on GammaPlan using the MD Anderson's IROC anthropomorphic SRS head phantom irradiation credentialing, 20 previously treated GK‐SRS patients (10 AVM, average volume 3.61 cm^3^ and 10 pituitary adenomas, average volume 0.86 cm^3^) who underwent manual forward planning on GammaPlan were retrospectively replanned via LDO. These pathologies were included because of the need for adequate dose delivery with organs at risk in very close proximity. LDO finds the target curvature boundary by well‐formulated linear programing objectives and inversely optimizes the GK‐SRS plan by isocenter placement, optimization, and sequencing. For identical target coverage, the LDO and original manual plans were compared for target conformity, gradient index, dose to critical organs, and surrounding normal brain. Additionally, various treatment delivery parameters, including beam‐on time were recorded.

**Results:**

For both patient cohorts, LDO provided similar target coverage with better dose conformity, tighter radiosurgical dose distribution with a lower value of gradient indices (all *p* < 0.001), and lower dose to critical organs. For AVMs, there was a significant reduction of normal brain *V*_10Gy_, *V*_12Gy_, and *V*_14Gy_ by 4.74, 3.67, and 2.67 cm^3^ (all *p* < 0.001). LDO had twice the number of shots (*p* < 0.001), and longer beam‐on time (*p* = 0.012) by a factor of 1.44. For pituitary adenomas, LDO provided systematically lower values of *V*_10Gy_, *V*_12Gy_, and *V*_14Gy_ by 1.08, 0.86, and 0.68 cm^3^ (all *p* < 0.001), and lower maximum dose to optic pathway by 0.7 Gy (*p* = 0.005), but had almost twice the numbers of shots (*p* < 0.001) and increased beam‐on time (*p* = 0.005) by a factor of 1.2. However, for both patient groups, the average planning time for the LDO was <5 min, compared to the estimated 30–90 min of manual planning times.

**Conclusion:**

GK‐SRS treatment on Leksell Perfexion GammaPlan using the LDO provided highly conformal target coverage with a steep dose gradient, spared critical organs, and significantly reduced normal brain dose for complex targets at the cost of slightly higher treatment times. LDO generated high‐quality treatment plans and could significantly reduce planning time. If available, the LDO algorithm is suggested for validation and clinical use for complex and difficult GK cases.

## 1 INTRODUCTION

Historically, the treatment planning of Gamma Knife (GK) stereotactic radiosurgery (SRS) patients required a manual forward planning approach by GK users (neurosurgeons, radiation oncologists, and medical physicists).^1^, ^2^, ^3^, ^4^, ^5^ The manual forward planning of the GK‐SRS plan could be very subjective, highly dependent on a planner's clinical experience, and could vary significantly in plan quality depending on irregular target shapes, and proximity to critical organs. Moreover, the 4, 8, 16‐mm collimators and blocking sectors are associated with an isocenter that has up to 65 535 possible shots configurations. This can make the decision of initial isocenter placement difficult even for an experienced user. Due to the benefit of same‐day SRS treatment procedures and because the patient has a metal headframe fixed on to their skull, generating a high‐quality treatment plan for the complex targets, in a short period of planning time, is highly desirable and patient friendly. Treatment planning of arteriovenous malformations (AVMs) is particularly challenging, due to the irregular shape of the target nidus, making it difficult to achieve a highly conformal dose distribution, a steep dose gradient, and spare the normal brain.^6^, ^7^ Similarly, due to the closed proximity of dose limiting critical organs, manually generating a highly conformal plan and sparing optic pathway (<8 Gy) in the treatment of pituitary adenomas is quite challenging and time‐consuming.^8^, ^9^ As mentioned earlier, the GK treatment planning process may take a significant amount of clinical time, with the manual placement of multiple shots and sectors beam blocking used to aid the target conformity while a patient is waiting for treatment with the metal headframe on. This may add significant pressure to the radiosurgery team in the delivery of high‐risk SRS treatment to AVMs and pituitary adenomas. Together, these two pathologies, in our view, represent the most challenging cases for safe and effective targeting with GK.

To automatically and efficiently generate a highly conformal GK‐SRS plan, Elekta Instrument AB, the manufacturer of the GK, recently developed a new fast inverse planning (FIP) module, called lightning dose optimizer (LDO), which uses a linear programing‐based dose optimization in Leksell GammaPlan (version 11.1.3).^10^ This LDO allows a planner to define a target, organs‐at‐risk (OARs) and to optimize the resulting dose distribution by prioritizing the low‐dose spread, beam‐on time, and limiting the maximum dose tolerances to the adjacent OARs. We have recently upgraded our GK Perfexion unit with the GammaPlan software (version 11.1.3) with LDO capability. The aim of this report is to quantify the dosimetric performance, treatment planning, and delivery times and initially evaluate LDO in Leksell GammaPlan software for delivering complex and difficult radiosurgery treatments for our most complex lesions—AVMs and pituitary adenomas.

## 2 MATERIALS AND METHODS

### 2.1 Independent dose verification

After upgrading the Leksell GK Perfexion, the clinical validation and implementation of the upgraded GammaPlan (version 11.1.3) software with an integrated LDO module for GK radiosurgery to intracranial lesions was performed by in‐house measurement and testing. Additionally, an independent dose rate verification of the Co‐60 sources and the updated GammaPlan software was completed by the end‐to‐end test using IROC MD Anderson's SRS credentialing anthropomorphic head phantom irradiation, containing a 1.9‐cm diameter spherical target and dosimetry systems (two orthogonal films and two thermoluminescent dosimeter [TLD] capsules) inserted. This phantom was imaged with Leksell Coordinate Frame G fixation (Elekta Instrument AB, Stockholm, Sweden), with both CT scan and MRI imaging, co‐registered those images in the GammaPlan, and an SRS treatment plan was generated for a single high‐dose of 25 Gy to the target for credentialing based upon the Alliance A071801 skull‐based brain SRS/SRT trial.^11^ Identical to a patient treatment procedure, the anthropomorphic head SRS phantom was then irradiated. Two TLD capsules provided dose information near the center of the target. Two orthogonal sheets of GAFChromic dosimetry media provided dose profiles and an evaluation of the delivered dose distribution. The credentialing results of IROC MD Anderson's SRS head phantom satisfied both the TLD and film dosimetric requirements established by the IROC for the SRS treatment on GK Perfexion unit with updated GammaPlan (version 11.1.3) with LDO capability. In this independent end‐to‐end test, the average absolute TLD dose ratio (planned vs. measured) and film measurement results were 1.00 and 97% gamma index over all three planes, respectively. The phantom irradiation results satisfied MD Anderson's credentialing requirement for SRS treatment using GammaPlan.

### 2.2 Patients population and contouring

After obtaining an Institutional Review Board approval from our institution, 20 previously treated GK‐SRS patients harboring single AVM (10 patients) and pituitary adenoma (10 patients) were included in this retrospective validation study. AVM contouring was performed in the GammaPlan software (version 11.1.3, Elekta Instruments AB) by an experienced neurosurgeon, by creating AP and lateral contours on stereotactic digitally subtracted angiography (DSA) images, then delineating the 3D shape of the nidus using gadolinium single‐contrast enhanced high‐resolution 1‐mm MRI images of the MPRAGE series that was co‐registered with the DSA images. The average target size derived from the DSA scan was 3.61 ± 2.05 cm^3^ (1.28–7.04 cm^3^). Pituitary adenoma contouring was done by the same neurosurgeon on the gadolinium single‐contrast enhanced high‐resolution 1‐mm MRI images, in the coronal view of the MPRAGE series. The mean tumor size was 0.86 ± 0.79 cm^3^ (0.24–2.84 cm^3^). For sparing the adjacent critical organs, optic apparatus was delineated in the coronal views as well.

### 2.3 Clinical manual treatment planning

The patient's plans were manually generated by expert GK users (greater than 5 years of GK planning experience at our GK center) and required the placing of multiple isocenter “shots” of different diameters into the target volume, in order to create a prescription isodose that conforms to the shape of the target. As described earlier, for GK Perfexion, each isocenter location has several thousand possible sector configuration combinations, which could make the GK planner's decision of initial isocenter placement a fairly difficult task to decide even for experienced users. For the AVMs, treatment plans were further optimized manually by adjusting the isocenter positions in 3D space, the relative weight of each isocenter, selecting an appropriate gamma angle, and the use of sector blocking that can also be used to enhance directional gradient to spare the adjacent critical organs, as desired. AVMs were prescribed 18–20 Gy at the 50% isodose line, with a maximum dose of 36–40 Gy inside the target per departmental clinical SRS protocol. For the pituitary adenomas, similar to AVMs, treatment plans were further optimized manually but always treated with 90° gamma angle and always used the appropriate manual sectors blocking that increased the dose gradient between the lesion and optic apparatus to maximum dose <8 Gy per our institutional standard. Prescription doses to pituitary adenomas were 18–28 Gy using the 50% isodose line, with a maximum dose of 36–56 Gy inside the lesion. Patients with nonfunctioning adenomas who underwent GK‐SRS received a mean target margin dose of 18 Gy, and those with Cushing disease received a mean margin dose of 24 Gy, with a maximum of up to 28 Gy in some cases.

### 2.4 Fast inverse planning via LDO

Patients were then replanned using updated GammaPlan software (version 11.1.3, Elekta Instruments AB) with an integrated LDO module for the Leksell GK Perfexion.^10^, ^12^ Briefly, the LDO performs the inverse planning in three stages utilizing a novel and well‐formulated linear objective function employing linear programing: isocenter placement, optimization, and sequencing.^12^ First, well‐distributed isocenter locations are generated in the target volume via two geometrical attributes skeleton and curvature of the target. The positions of the isocenters are then unchanged in the subsequent optimization steps. Second, an optimization problem is formulated as the weighted sum of all the objectives and constraints by ensuing in a cost function that utilizes the target dose coverage, and sparing the OARs penalties by high selectivity and high‐dose gradient. Finally, LDO works by penalizing the beam‐on time during optimization: times for each collimator are minimized but allowed to vary independently and are then converted to deliverable shots in the final sequencing phase of the plan optimization.

For the identical prescription dose and isodose line, the LDO plans were generated to match or exceed the manual plan quality metrices for the target coverage, Paddick conformity index (PCI), gradient index (GI),^13^, ^14^ and minimized the maximum dose to the adjacent OARs. The input parameters for the LDO plan include the prescription dose, maximum target dose, adjustable low‐dose and beam‐on time penalties, and maximum dose tolerances to each critical organ. The LDO plans were generated within a few seconds. If the desired goals were not achieved, the user is allowed to adjust the low‐dose spread and beam‐on time penalties and generate a new plan for comparison. A new LDO plan can be generated in a few seconds as well. For instance, for AVM, the initial LDO plan was executed with 50/50 (range, 0–100) optimization settings for the low‐dose spread and beam‐on time priorities on the optimizer window. This was followed by a successive optimization of the parameters setting by adjusting the optimal target coverage, dose conformity, and low‐dose spared the adjacent critical organs. However, due to the relatively smaller size of the pituitary adenoma and the proximity of the dose limiting OARs (optic apparatus), the LDO was performed with 50/50 (range, 0–100) optimization setting for the low‐dose spread and 30/70 (range, 0–100) setting on beam‐on time penalties on the LDO window in GammaPlan and maximum dose limit to optic apparatus was set to less than 8 Gy.

### 2.5 Plan evaluation

Per our clinical standard, for both patient cohorts, both clinical manual forward and LDO plans were prescribed to 50% isodose line and identical maximum dose to the target. Treatment times were recalculated for a common dose rate for a pair of plans. The isodose distributions, dose–volume histograms (DVHs), and target dose metrics of the manual and the fast inverse LDO plans were evaluated following the QUANTEC^8^ and our institutional SRS protocol guidelines. Additionally, these plans were evaluated for target conformity by PCI and Paddick GI^12^, ^13^ as mentioned earlier. The maximum doses to optic apparatus, brainstem, and normal brain receiving *V*_10Gy_, *V*_12Gy_, and *V*_14Gy_, respectively, were evaluated. Furthermore, treatment planning time was estimated for both manual forward and LDO planning. Beam‐on time was documented. To assess the normality of each parameter, the Shapiro test followed by an evaluation of skewness and kurtosis was conducted. For both patient cohorts, a comparison of dosimetric parameters and plan quality metrics were performed using the Wilcoxon rank *t*‐test (nonparametric) between the manual forward and fast inverse LDO plans for a significance level of the *p*‐value of <0.05.

## 3 RESULTS

### 3.1 Arteriovenous malformations

For AVM, plan quality and target metrics are displayed in Table 1 for both clinical manual and LDO plans, each demonstrating compliance with our departmental SRS protocol standard. Compared to manual clinical plans, LDO plans showed systematically better tumor conformity and dose gradient as demonstrated by the values of PCI and GI. This signifies a superior tighter 25% isodose distribution with LDO compared to the clinical manual plans.

**TABLE 1:** Evaluation of target coverage metrics, treatment delivery parameters, and normal brain *V*_10_, *V*_12_, and *V*_14_ for all arteriovenous malformation (AVM) cases

Target volume (cm^3^)	3.61 ± 2.05 (1.28–7.04)		
Parameters	Forward manual plan	Inverse LDO^a^ plan	*p* *‐Value*
PCI^b^	0.59 ± 0.07 (0.46–0.71)	0.75 ± 0.05 (0.69–0.83)	*<0.001*
Paddick GI^c^	3.04 ± 0.14 (2.81–3.32)	2.54 ± 0.11 (2.37–2.64)	*<0.001*
Number of shots used	18 ± 7 (10–30)	37 ± 14 (13–58)	*<0.001*
BOT^d^ (min)	69.4 ± 23.2 (41.0–105.0)	95.7 ± 32.5 (45.0–171.3)	*=0.012*
*V*_10Gy_ (cm^3^)	15.77 ± 6.08 (7.50–25.90)	11.04 ± 4.57 (5.70–17.90)	*<0.001*
*V*_12Gy_ (cm^3^)	12.23 ± 4.77 (5.90–19.60)	8.56 ± 3.42 (4.50–14.00)	*<0.001*
*V*_14Gy_ (cm^3^)	9.85 ± 3.89 (4.80–15.30)	7.18 ± 3.06 (3.70–13.10)	*<0.001*

Prescription dose was 18–20 Gy, prescribed to 50% isodose line to the surface of the target. Mean ± standard deviation (SD) (range) was reported.

^a^Lightning dose optimizer.

^b^Paddick conformity index.

^c^Gradient index.

^d^Beam‐on time.

Major dosimetric differences (mean and standard deviation) between clinical manual and LDO plans in terms of normal brain *V*_10Gy_, *V*_12Gy_, and *V*_14Gy_ were observed (see Table 1). Statistically significant differences (all *p* < 0.001) were found for all of the evaluated dosimetric parameters relevant to patient care with a clear trend of significantly decreased dose to normal brain with LDO plans (see highlighted *p*‐values). For the same target coverage, the average reduction of *V*_10Gy_, *V*_12Gy_, and *V*_14Gy_ by 4.74, 3.67, and 2.67 cm^3^, respectively, via LDO could have a clinically significant response in treatment outcomes. However, LDO plans had as many as twice the number of shots (*p* < 0.001) and relatively longer beam‐on time (*p* = 0.012) by a factor of 1.44, on average, compared to the manual plans (Table 1).

Due to the retrospective nature of this validation study, the overall treatment planning times for the clinical manual plans were not recorded in the past but are estimated to vary from 30 to 90 min based on the complexity of the target and the clinical experience of the GK planner. When compared to LDO plans, the plan optimization time is drastically reduced with the use of the fast inverse optimizer. Overall, the LDO was shown to take an average of two to three iterations with a mean optimization time of 3 min for inversely optimized plans (range: 1.0–8.0 min)—showing a lower treatment planning time for each AVM case.

**FIGURE 1:**
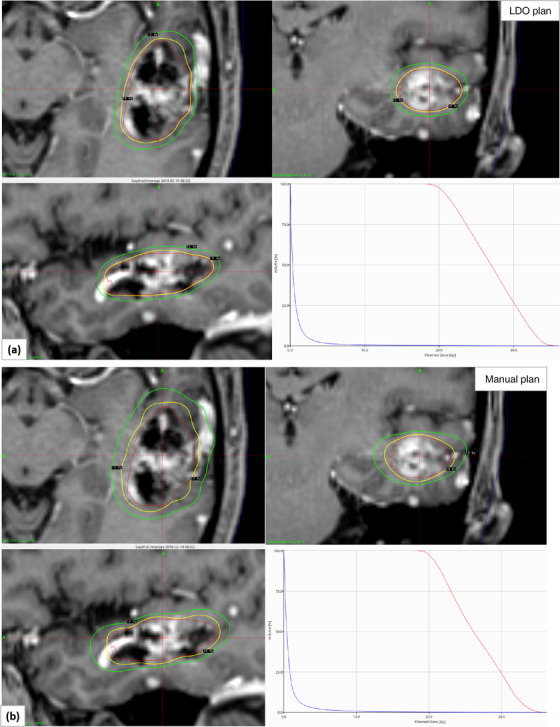
Demonstration of the axial, coronal, and sagittal views of isodose distribution (yellow, 18‐Gy prescription and green, 12‐Gy isodose lines) in the post‐contrast MPRAGE MRI images showing the difference between the lightning dose optimizer (LDO) (a, upper panel) and manual clinical plan (b, lower panel) of the representative case with the major improvement with the new inverse planning optimizer. The graphs show the dose–volume histogram (DVH) plot for nidus (red) and body contour (blue). LDO improved conformity and dose gradient and significantly reduced normal brain *V*_10Gy_, *V*_12Gy_, and *V*_14Gy_ by 6.4, 6.7, and 5.5 cm^3^, respectively.

Figure 1 shows a radiosurgical dose distribution in the axial, coronal, and sagittal views for a patient who underwent AVM treatment in the left temporal lobe that was replanned with LDO (a, upper panel) compared to the original clinically delivered manual plan (b, lower panel). Much tighter and clinically desirable 50% prescription isodose lines and tighter *V*_12Gy_ were obtained with LDO (see green isodose line) compared to the original clinical manual plan. DVH parameters are shown for the target coverage, dose gradient, and normal brain dose for clinical manual versus LDO plans, suggesting that a dosimetrically superior SRS treatment plan was obtained with the new FIP algorithm in GammaPlan. The target size was 6.5 cm^3^. This is a relatively large tumor size in this cohort and is located in the left temporal lobe. In this case, for the same target coverage, the PCI, GI, and normal brain *V*_10Gy_, *V*_12Gy_, *V*_14Gy_ were 0.83 versus 0.67, 2.38 versus 2.95, 16.5 versus 22.9 cm^3^, 11.5 versus 17.9 cm^3^, and 9.1 versus 14.6 cm^3^ for LDO versus clinical manual plan, respectively—all parameters favoring the LDO plan. However, in this case, the inverse optimizer provided 55 shots and a relatively longer beam‐on time of 108 min compared to a total of 22 shots and 59 min of a beam‐on time with the original manual clinical plan.

### 3.2 Pituitary adenomas

For pituitary adenomas, plan quality and target metrics are displayed in Table 2 for both the original manual and LDO plans, each demonstrating compliance with our departmental SRS guidelines. Compared to manual clinical plans, LDO showed better tumor conformity, and a steeper dose gradient as shown by PCI and GI, showing systematically superior plan quality for all patient's plans—also demonstrating a lower treatment planning time for each pituitary case.

**TABLE 2:** Evaluation of target coverage metrics, treatment delivery parameters, normal brain *V*_10Gy_, *V*_12Gy_, and *V*_14Gy_, and maximum dose to optic apparatus for all pituitary adenoma cases

Target volume (cm^3^)	0.86 ± 0.79 (0.24–2.84)		
Parameters	Forward manual plan	Inverse LDO^a^ plan	*p*‐Value
PCI^b^	0.51 ± 0.11 (0.36–0.66)	0.63 ± 0.08 (0.51–0.73)	*<0.001*
Paddick GI^c^	3.06 ± 0.41 (2.49–3.89)	2.69 ± 0.16 (2.41–2.92)	*=0.006*
Number of shots used	18 ± 8 (9–34)	24 ± 9 (15–42)	*<0.001*
BOT^d^ (min)	107.9 ± 26.4 (66.0–166.0)	128.9 ± 27.8 (93.6–194.4)	*=0.005*
*V*_10Gy_ (cm^3^)	4.86 ± 2.14 (3.00–10.60)	3.78 ± 1.91 (1.90–9.70)	*<0.001*
*V*_12Gy_ (cm^3^)	3.83 ± 1.70 (2.50–8.40)	2.97 ± 1.53 (1.50–6.90)	*<0.001*
*V*_14Gy_ (cm^3^)	3.11 ± 1.41 (1.90–6.90)	2.43 ± 1.27 (1.20–5.70)	*<0.001*
Max dose to optic apparatus (Gy)	6.50 ± 1.32 (3.50–8.00)	5.82 ± 1.05 (3.20–7.00)	*=0.005*

Prescription was 18–28 Gy, prescribed to 50% isodose line to the surface of the pituitary tumor. Mean ± standard deviation (SD) (range) was reported.

^a^Lightning dose optimizer.

^b^Paddick conformity index.

^c^Gradient index.

^d^Beam‐on time.

The major dosimetric differences (mean, standard deviation, and range) between clinical manual and LDO plans in terms of normal brain sparing *V*_10Gy_, *V*_12Gy_, and *V*_14Gy_ and maximum dose to optic apparatus were observed (see Table 2). Statistically significant differences (all *p* < 0.001) were found for all of the evaluated dosimetric parameters with a stronger trend of significantly decreased dose to normal brain and optic pathway with LDO plans (see, emboldened *p*‐values). For the same target coverage, the average of *V*_10Gy_, *V*_12Gy_, *V*_14Gy_ and maximum dose to optic pathway was reduced by 1.08, 0.86, 0.68 cm^3^, and 0.7 Gy, respectively, using LDO. However, LDO plans provided almost twice the number of shots (*p* < 0.001) and relatively longer beam‐on time (*p* = 0.005) by a factor of 1.2, on average, compared to the manual clinical plans (Table 2). Similar to the AVM cases, due to the retrospective nature of this validation study, the overall treatment planning times for prior clinical manual plans were not recorded, but estimated to vary from 30 to 90 min based on the complexity of the target, proximity of the optic apparatus, and the clinical experience of the GK planner. When compared to LDO plans, the plan optimization time was significantly reduced with the use of the new inverse optimizer. Overall, these plans can be optimized in three iterations, on average, with a mean optimization time of 5 min for LDO plans (range: 3–12 min)—significantly reducing the GK planning time and potentially improving the clinic workflow.

**FIGURE 2:**
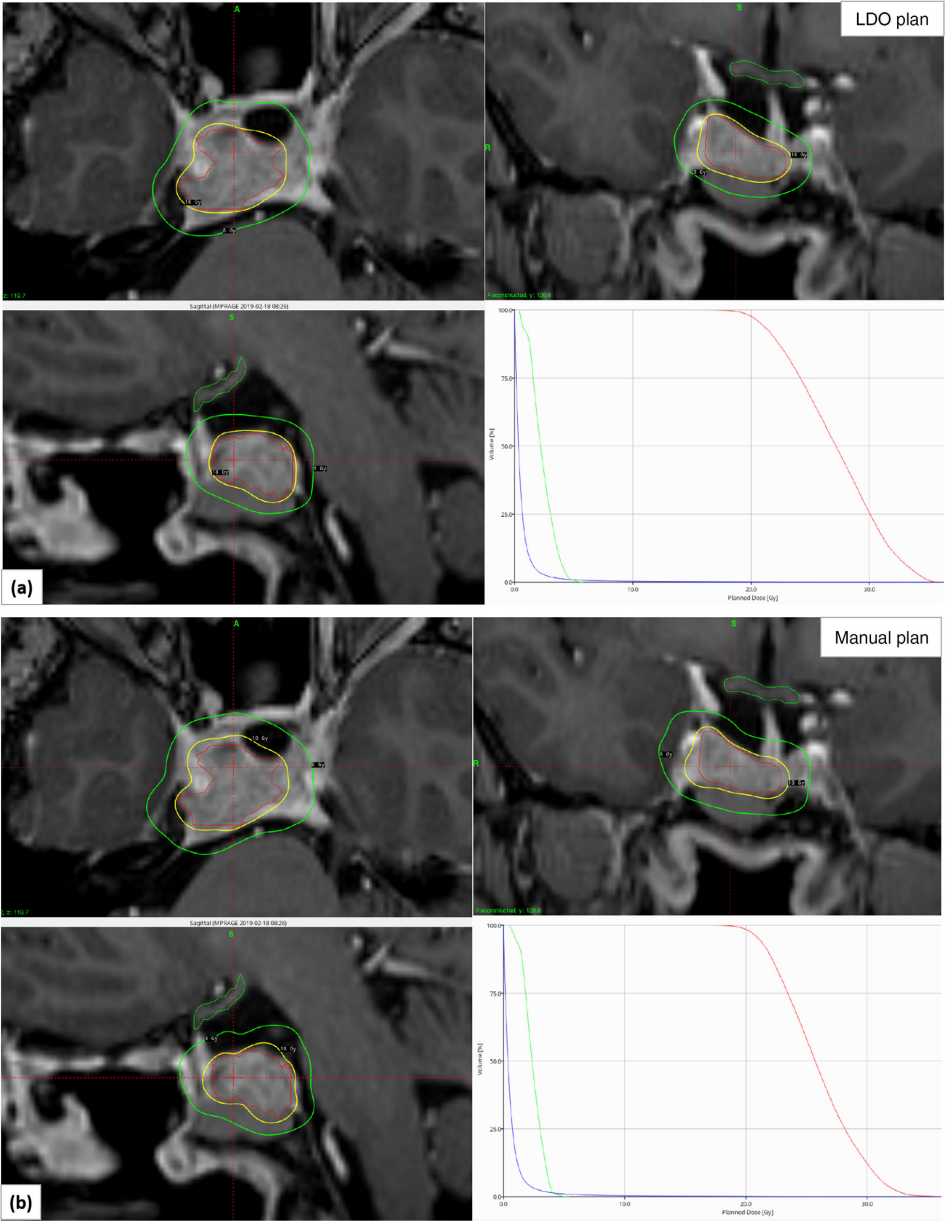
Axial, coronal, and sagittal views of isodose distribution (yellow, 18 Gy prescription and green, 8 Gy isodose lines) in the post‐contrast MPRAGE MRI images demonstrating the dosimetric differences between the lightning dose optimizer (LDO) (a, upper panel) and manual clinical plan (b, lower panel) of the representative pituitary cancer patient with the major improvement of the plan quality via new fast optimizer. The graphs show the dose–volume histogram (DVH) plot for pituitary adenoma (red), optic apparatus (green), and body contour (blue). Compared to the manual plan, LDO improved conformity and dose gradient and significantly reduced normal brain *V*_10Gy_, *V*_12Gy_, *V*_14Gy_, and maximum dose to optic apparatus by 1.9, 1.5, 1.2 cm^3^, and 1.9 Gy, respectively.

Figure 2 shows an example case of radiosurgical dose distribution in the axial, coronal, and sagittal views for a patient who presented with a pituitary tumor replanned with LDO (a, upper panel) and the original clinical manual plan (b, lower panel). Much tighter 50% prescription isodose radiosurgical distribution and a tighter *V*_8Gy_ were obtained with LDO (see green isodose line) compared to the original clinical manual plan. DVH parameters are shown for the tumor coverage, steep dose gradient, and dose to optic apparatus for clinical manual versus LDO plan—suggesting that a dosimetrically superior plan quality was obtained with the new approach. The tumor size was 2.84 cm^3^. This is a relatively large tumor size in this patient cohort and is located medially. In this case, for the same target coverage, the PCI, Paddick GI, normal brain *V*_10Gy_, *V*_12Gy_, *V*_14Gy_, and maximum dose to optic apparatus were 0.73 versus 0.58, 2.50 versus 2.54, 8.70 versus 10.60 cm^3^, 6.90 versus 8.4 cm^3^, 5.7 versus 6.9 cm^3^ and 5.1 versus 7.0 Gy for LDO versus clinical manual plan, respectively—systematically all parameters pointed to a preference for the inversely optimized plan. However, in this case, the LDO plan provided a relatively larger number of shots (42), but a similar beam‐on time of 135 min compared to a total number of shots (29) with 132 min of a beam‐on time with the manual clinical plan.

## 4 DISCUSSION

In this study, we have presented the clinical validation and implementation of the new FIP algorithm via LDO on Leksell Perfexion GammaPlan for a stereotactic treatment of complex and irregular targets such as AVM and pituitary adenomas. Following the independent dose verification by IROC MD Anderson's SRS head phantom irradiation and credentialing results, we found that all plans generated via LDO for the complex and difficult targets of AVM and pituitary adenomas had a higher target conformity, and rapid dose fall‐off around the target. All pituitary plans met the optic apparatus dose tolerances limit of less than 8.0 Gy per our departmental SRS protocol requirement. It should be noted that the steep dose gradient around the target with LDO plans along with an average value of GI of <2.7 was highly desirable for SRS treatment in both patient cohorts (Tables 1 and 2). Moreover, the main advantages of the LDO plans were a significant reduction of normal brain irradiation demonstrated by systematically low values of normal brain *V*_10Gy_, *V*_12Gy_, and *V*_14Gy_ (Tables 1 and 2) with a significantly shorter GK planning times (average <5 min) compared to manual forward planning (average 30–90 min). However, the total number of shots and overall beam‐on time were increased LDO in both patient cohorts.

As mentioned earlier, historically GK‐SRS plans were generated manually by the GK users. The plan quality is heavily dependent on planner's clinical experience and available treatment planning time between the MRI imaging or CT scan times and starting of the patient's treatment. In our experience, to generate a clinically acceptable GK‐SRS plan for complex and difficult targets such as AVM or pituitary adenomas takes about 30–90 min of dedicated planning time for an experienced planner. Delaying treatment start time could cause headframe movement, longer patient waiting time could cause discomfort and anxiety and could potentially lose the effectiveness of local anesthesia—perhaps leading to the patient having a headache and additional pain in some instances. In the past, Leksell GammaPlan released a commercially available inverse planning algorithm.^5^ However, due to the challenging nature of the non‐convex inverse optimization problem, the inverse planning algorithm required much more manual adjustments after plan optimization and did not do well clinically. To the best of our knowledge, it did not achieve widespread clinical utilization in the GK radiosurgery community, whereas the new LDO algorithm provided a clinically acceptable plan within a few minutes of planning times. Moreover, potential concerns of normal brain necrosis and radiation‐induced toxicity in the treatment of AVM and pituitary adenomas are reported by several researchers.^6^, ^7^, ^9^, ^15^, ^16^ According to QUANTEC guidelines,^8^ *V*_12Gy_ brain volume predicts radiation‐induced necrosis, where the risk of toxicity increases from 23% for *V*_12Gy_ between 0 and 5 cm^3^ to 54% for *V*_12Gy_ between 10 and 15 cm^3^ in the treatment of brain lesions. To overcome this problem and to efficiently generate acceptable GK‐SRS plans that maximize the target conformity while minimizing dose GI and dose to surrounding organs, Leksell GammaPlan implemented a novel linear programing‐based algorithm that we found is simple to implement in the GK center and clinically useful for patients while significantly sparing the normal brain tissue that could potentially translate to favorable clinical outcomes in terms of reducing treatment‐related toxicity in the future.

In the past, a few researchers have studied the inverse planning approach for GK‐SRS cases.^17^, ^18^, ^19^ For instance, Paddick et al.^17^ evaluated the dosimetric performance of a commercially released IntuitivePlan inverse planning approach for GK radiosurgery of AVMs. When compared to manual forward plans, the IntuitivePlan resulted in a larger number of shots, similar to our study. Similarly, Levivier and colleagues^18^ integrated standalone IntuitivePlan software into the GammaPlan via exporting and importing patient datasets and provided similar results of manual planning with a relatively larger number of shots and longer beam‐on time, similar to this study. In contrast to this offline planning software, LDO is implemented in the GammaPlan platform, hence, is much more time efficient. At the time of this manuscript preparation, Wieczorek and colleagues^20^ published a paper in the Medical Dosimetry Journal showing the plan quality metrices of the Leksell GK LDO for multiple sites of the brain disease, including AVM's. They have reported the target coverage, conformity, GI, number of shots, and beam‐on time; however, no dose to normal brain and dose to critical organs were presented. Our study complements their report by independently validating LDO and rapidly generating the high‐quality GK‐SRS plans for the irregular, complex, and difficult targets such as AVMs and pituitary adenomas.

Preplanning capability for the GK‐SRS using previously obtained MRI images has been available on GammaPlan and most recently the preplanning clinic workflow was reported for the prospective patients on LGK Icon.^21^, ^22^ However, in our current clinic workflow with LGK Perfexion, we do not use the preplanning capability. Nevertheless, for all plans generated with LDO, the planning time was remarkably shorter than with manual planning and lower dose to OARs, including normal brain. For the complex and irregular target volume that is close to the critical organs, we still need an expert planner to further review and adjust the optimization parameters on LDO, re‐optimize the plan quickly, and assess the plan deliverability metrics to achieve the final optimal clinical plan.

Limitations of this study include its retrospective nature of the GK‐SRS planning and clinical validation study. Nevertheless, we are encouraged by the results of this validation study and have started using LDO clinically for the complex AVM and pituitary adenomas plans. Further evaluation of LDO's performance, including large tumors, irregular resection cavities,^23^ irregular‐shaped acoustic neuromas,^24^ large and irregular meningiomas,^25^ and a large number of multiple brain metastases,^26^, ^27^ is ongoing in our historic GK radiosurgery center.

## 5 CONCLUSION

Independent dose verification of the LDO module in Leksell GammaPlan by IROC MD Anderson's SRS head phantom irradiation met the credentialing requirements for SRS treatment. For irregular, complex, and difficult targets such as AVM and pituitary adenomas, LDO provided highly conformal dose distribution, rapid dose fall‐off around the target, excellent sparing of adjacent critical organs such as optic apparatus and significantly reduced normal brain irradiation. Even though plans have a higher number of shots and relatively longer beam‐on time, due to the better plan quality, lower normal brain dose, and very short planning time, we have clinically implemented LDO for complex and irregular targets plan optimization in our radiosurgery center.

## CONFLICT OF INTEREST

All coauthors have no conflict of interest to declare.

## AUTHOR CONTRIBUTIONS

Damodar Pokhrel, PhD, conceptualized the project, generated gamma knife radiosurgery treatment plans, and collected and analyzed the data. Justin F. Fraser, MD, provided neurosurgery expertise and supervision of the project. Mark E. Bernard, MD, James Knight II, MD, and William St. Clair, MD, PhD, provided radiation oncology clinical expertise and supervision of the paper. Damodar Pokhrel, PhD, outlined and drafted the preliminary manuscript and all coauthors revised and approved the final manuscript for submission.

## Data Availability

All data are available upon request only due to privacy/ethical restrictions.
